# Nominal Concentration-Based Assessment of Structure-Dependent PFAS Responses in the Freshwater Diatom *Cyclotella meneghiniana* Using Dual Physiological Endpoints

**DOI:** 10.3390/jox16040155

**Published:** 2026-08-21

**Authors:** Hojun Lee, Taejun Han, Jihae Park

**Affiliations:** 1 Marine@UGent Korea, Ghent University Global Campus, 119-5, Songdomunhwa-ro, Incheon 21985, Republic of Korea; hojun.lee@ugent.be (H.L.); taejun.han@ghent.ac.kr (T.H.); 2 Bio Environmental Science and Technology (BEST) Lab, Ghent University Global Campus, 119-5, Songdomunhwa-ro, Incheon 21985, Republic of Korea; 3 Department of Animal Sciences and Aquatic Ecology, Ghent University, Coupure Links 653-Block F, B-9000 Gent, Belgium; 4 Center for Green Chemistry and Environmental Biotechnology, Ghent University Global Campus, 119-5, Songdomunhwa-ro, Incheon 21985, Republic of Korea

**Keywords:** Per- and polyfluoroalkyl substances, diatom bioassay, chlorophyll fluorescence, lipid accumulation

## Abstract

Per- and polyfluoroalkyl substances (PFAS) are persistent, structurally diverse contaminants whose ecological hazard assessment remains challenging, particularly for primary producers. Conventional growth-based algal bioassays often overlook sublethal responses associated with chronic exposure. Here, we developed a 7-day multigenerational bioassay using the freshwater diatom *Cyclotella meneghiniana* that integrates chlorophyll fluorescence (photosynthetic biomass) and lipid accumulation (oxidative stress and carbon reallocation) as complementary endpoints. Ten PFAS spanning carbon chain lengths from C4 to C11 and representing both perfluoroalkyl carboxylates and sulfonates were evaluated under identical nominal exposure conditions. Distinct structure-dependent toxicity patterns emerged. Long-chain perfluoroalkyl carboxylic acids elicited the strongest responses, whereas short-chain compounds produced no measurable effects. Among the sulfonates, PFHxS reduced chlorophyll fluorescence with limited effects on lipid accumulation, whereas PFOS produced no measurable response. Exploratory species sensitivity distribution analyses indicated compound-specific differences, although predicted no-effect concentrations should be interpreted cautiously because they combine literature-derived growth endpoints with endpoint-specific values generated in this study. This dual-endpoint diatom bioassay provides a proof-of-concept platform for structure-informed PFAS hazard ranking rather than regulatory toxicity assessment.

## 1. Introduction

Per- and polyfluoroalkyl substances (PFAS) comprise a vast and structurally diverse group of synthetic chemicals that are widely used in industrial and consumer products, including firefighting foams, non-stick coatings, textiles, and food packaging [1]. Their defining feature includes the exceptional stability of the carbon–fluorine bond, which renders them highly resistant to environmental degradation, leading to their persistence and widespread distribution across surface waters, sediments, and biota, even in remote ecosystems [2]. Consequently, they are frequently called “forever chemicals,” with mounting evidence linking chronic exposure to adverse health outcomes, including endocrine disruption, immunotoxicity, metabolic disorders, and carcinogenic effects [3].

Despite growing concern, regulatory frameworks address only a limited portion of the broader PFAS chemical space. For example, the U.S. EPA’s 2024 National Primary Drinking Water Regulation established standards for PFOA, PFOS, PFHxS, PFNA, and HFPO-DA, together with a hazard-index approach for selected PFAS mixtures [4]. Internationally, the Stockholm Convention lists PFOS, PFOA, PFHxS, and, since 2025, long-chain perfluorocarboxylic acids and their related compounds [5]. The European Union has also established a surface-water quality standard of 0.65 ng L^−1^ for PFOS [6]. Nevertheless, thousands of structurally diverse PFAS remain unregulated and toxicologically insufficiently characterized, posing a major challenge for environmental risk assessment [7,8]. This challenge is further complicated by the structural diversity of PFAS, which encompass a continuum of compounds with differing physicochemical properties, environmental behaviors, and biological effects, making structure-informed assessment approaches increasingly necessary. This structural diversity includes variation in perfluorinated carbon-chain length and terminal functional groups, such as carboxylates and sulfonates, which contribute to differences in aqueous solubility, environmental mobility, sorption and bioaccumulation potential, as well as biological interactions and toxicity [9,10].

Current PFAS toxicity assessments are largely based on vertebrate models and rely on endpoints such as survival, growth, and reproduction, which form the basis of regulatory benchmarks [10,11,12]. While these endpoints are essential, they largely overlook potential impacts on lower trophic levels. Primary producers, particularly photosynthetic microorganisms, form the foundation of aquatic food webs and are directly exposed to dissolved contaminants. Sublethal perturbations at this level can propagate through trophic interactions, ultimately affecting ecosystem structure and function [13].

In parallel, ecotoxicological bioassays have emerged as critical tools for assessing biological responses that are not captured by chemical analyses alone. Standardized assays based on growth inhibition in green algae and cyanobacteria (e.g., ISO 8692; OECD 201) are widely used in regulatory frameworks [14,15]. However, these assays primarily measure acute responses and focus on growth endpoints, potentially overlooking sublethal effects that develop over longer exposure periods. This limitation is particularly relevant for PFAS, which often exhibit low acute toxicity while inducing chronic physiological disturbances that may not be detected in short-term assays. Such disturbances may include impaired photosynthetic function, oxidative imbalance, altered energy metabolism, and changes in lipid metabolism.

Diatoms, a major group of microalgae characterized by their siliceous cell walls, play a central role in global biogeochemical cycles and contribute approximately 20% of global primary production [16]. Their ecological importance, combined with their sensitivity to environmental stressors, makes them highly relevant model organisms for ecotoxicological studies [17]. However, despite this importance, the chronic sublethal responses of diatoms to emerging contaminants such as PFAS remain comparatively understudied [18,19]. Previous studies have investigated PFAS toxicity in several freshwater and marine microalgae using growth, photosynthetic, oxidative-stress, and molecular endpoints. For example, PFOS-induced oxidative damage and cytotoxicity have been examined in *Chlorella vulgaris* [20], while PFOS and PFBS were shown to affect photosynthetic characteristics, oxidative-stress responses, and gene expression in *Scenedesmus obliquus* [21]. PFOA-induced effects on growth and cellular physiology have also been investigated in freshwater green microalgae [22,23], and more recent studies have extended PFAS assessment to marine *Chlorella* [24] and the diatoms *Thalassiosira pseudonana* [25] and *Nitzschia palea* [19]. In addition, transcriptomic responses to a broader panel of 22 PFAS have recently been evaluated in *Raphidocelis subcapitata* [26]. Collectively, these studies demonstrate that PFAS can affect multiple aspects of microalgal physiology and also show the recent expansion of PFAS ecotoxicology beyond conventional green microalgal models to diatoms. Nevertheless, systematic studies comparing multiple structurally diverse PFAS in freshwater diatoms using complementary physiological endpoints remain limited.

*Cyclotella meneghiniana* was therefore selected as a freshwater diatom model that complements the green microalgae more commonly used in freshwater ecotoxicology. As a diatom, it represents a phylogenetically and physiologically distinct lineage of primary producers, thereby broadening the biological representation of freshwater microalgae in PFAS toxicity assessment. It also has practical and physiological advantages for the chronic multi-endpoint design used here. Its relatively rapid growth allows a 7-day exposure to encompass approximately 5–8 generations, providing a multigenerational exposure window within a practical laboratory test period. Moreover, its photosynthetic physiology and capacity for lipid accumulation enable chlorophyll-associated and metabolic responses to be assessed within the same organism. Thus, *C. meneghiniana* was not selected on the assumption that it is intrinsically more sensitive or superior to standard green-algal test species, but because it combines complementary freshwater diatom representation, suitability for multigenerational exposure, and compatibility with two physiologically distinct endpoints. These characteristics make it particularly suitable for the systematic comparison of structure-dependent responses across the diverse PFAS panel examined in this study.

Mechanistic studies suggest that PFAS exposure disrupts key cellular processes, including mitochondrial function, oxidative balance, and energy metabolism [27]. In various microalgal species, PFAS have been shown to impair photosynthetic electron transport, induce oxidative stress, and increase reactive oxygen species (ROS) production, leading to lipid peroxidation and reduced photosynthetic efficiency [20,21,24]. These findings point to a conserved stress-response cascade in which early reductions in photosynthetic pigment content and biomass are followed by downstream metabolic adjustments. Photosynthesis and lipid metabolism are metabolically coupled because photosynthetic carbon fixation and energy generation provide substrates and reducing power for lipid biosynthesis; under physiological stress, disruption of cellular carbon and energy balance can alter carbon allocation and promote the accumulation of storage lipids such as triacylglycerols. This metabolic coupling provides the biological rationale for evaluating chlorophyll fluorescence and lipid accumulation as complementary physiological endpoints [28,29].

To capture these temporally structured responses, there is a need for bioassays that integrate endpoints representing different stages of the stress pathway. Bulk chlorophyll fluorescence (measured here as steady-state emission at Ex 485/Em 680 nm) provides an integrated signal reflecting chlorophyll content and photosynthetic pigment status per culture volume, enabling detection of early reductions in photosynthetic biomass and pigment content. Lipid accumulation, commonly measured using Nile Red staining, reflects downstream metabolic responses associated with oxidative stress and carbon reallocation [28,29,30]. Together, these mechanistically linked but temporally distinct endpoints provide a means to resolve the progression of cellular stress. Compared with alternative endpoints such as ROS detection or enzyme assays, they offer improved reproducibility and compatibility with standardized testing frameworks [31,32]. Despite these advantages, bioassays that integrate mechanistically distinct endpoints within a chronic exposure framework remain underdeveloped, particularly for diatom species.

The aim of the present study was to develop a chronic multi-endpoint bioassay framework for comparing complementary physiological responses to PFAS exposure beyond those captured by conventional single-endpoint acute tests. We applied this framework systematically across a structurally diverse PFAS panel using the freshwater diatom *Cyclotella meneghiniana*, a species not previously used as a test organism in PFAS ecotoxicology. By integrating chlorophyll fluorescence and lipid accumulation, the framework was designed to capture complementary photophysiological and metabolic responses within a single chronic assay. Importantly, the objective of this work was to compare the relative biological sensitivity of structurally diverse PFAS under identical exposure conditions, rather than to derive regulatory toxicity thresholds for individual compounds.

We hypothesized that chronic physiological responses in *C. meneghiniana* would be structure dependent, with longer-chain PFAS generally eliciting stronger effects, while the magnitude and pattern of toxicity would also vary with terminal functional group. We further hypothesized that these structure-dependent effects would be expressed differently between chlorophyll fluorescence and lipid accumulation, reflecting the distinct but complementary photophysiological and metabolic processes represented by the two endpoints.

## 2. Materials and Methods

### 2.1. Diatom Cultivation

The freshwater diatom *Cyclotella meneghiniana* was cultured in diatom medium (DM; [33] at 20 ± 1 °C under continuous white LED illumination (30 ± 2 μmol photons m^−2^ s^−1^) with a 12 h light–12 h dark photoperiod. To prevent sedimentation, cultures were agitated gently once daily.

### 2.2. Toxicity Testing

#### 2.2.1. Preparation of PFAS Solutions

Ten PFAS compounds were selected to represent key structural and regulatory groups, encompassing carbon chain lengths from C4 to C11 and both carboxylate or sulfonate functional groups (Table 1). Selection was guided by their relevance to national monitoring programs in South Korea [34] and to international regulatory frameworks, including the Stockholm Convention and EU/US EPA standards [5].

All chemicals were of analytical grade and obtained from Sigma-Aldrich (St. Louis, MO, USA). Perfluorobutanoic acid PFBA, perfluorohexanoic acid (PFHxA), perfluorooctanoic acid (PFOA), perfluorononanoic acid (PFNA), and perfluorooctane sulfonate (PFOS) were dissolved in distilled water. Perfluoropentanoic acid (PFPeA), perfluoroheptanoic acid (PFHpA), and perfluorohexane sulfonate (PFHxS) were dissolved in dimethyl sulfoxide (DMSO; ≥99.9%), and perfluorodecanoic acid (PFDA) and perfluoroundecanoic acid (PFUnDA) were dissolved in acetone (≥99.9%).

The final solvent concentration in all test solutions was maintained below 0.1% (*v/v*) to minimize solvent-related effects. Stock solutions were prepared fresh prior to each experiment. All test solutions were adjusted to pH 6.9 ± 0.2 using 1 M NaOH or 1 M HCl. Water hardness and conductivity were not independently monitored during the exposure period. Because all treatments were prepared using the same defined diatom medium, the nominal ionic composition of the medium was consistent across treatments. For each compound, five nominal test concentrations were prepared in DM. The nominal concentrations were 62.5, 125, 250, 500, and 1000 mg L^−1^ for PFBA, PFPeA, PFHxA, PFOA, and PFNA; 15.625, 62.5, 125, 250, and 500 mg L^−1^ for PFHpA, PFHxS, and PFOS; 0.625, 1.25, 2.5, 5, and 10 mg L^−1^ for PFDA; and 0.25, 0.5, 1, 2, and 4 mg L^−1^ for PFUnDA. The highest nominal concentration was determined based on the aqueous solubility of each compound (Table 1).

Because the present study was designed to compare the relative biological sensitivity of structurally diverse PFAS under identical exposure conditions, rather than to determine regulatory toxicity thresholds for individual compounds, nominal concentrations were used throughout. All exposure solutions were freshly prepared from analytical-grade standards immediately before each test; identical vessels, working volumes, and handling procedures were used for every treatment; the exposure duration was fixed at 7 days for all compounds; and all compounds were tested in the same medium and under the same temperature and light conditions. Because dissolved PFAS concentrations were not analytically verified, the actual aqueous exposure concentrations cannot be determined from the present study. Compound-specific losses through adsorption to test vessels, partitioning, or other processes may introduce uncertainty into both absolute EC_50_ values and comparative rankings. Dissolved-phase concentrations may differ from nominal values, particularly for long-chain PFAS. Nominal concentrations were used to provide a standardized basis for the initial comparative screening of multiple PFAS under identical experimental conditions and should not be interpreted as analytically verified dissolved exposure concentrations. Accordingly, the reported EC_50_ values represent nominal exposure–response metrics, and analytical verification will be required for quantitative toxicity-threshold or regulatory interpretation. Accordingly, the EC_50_ values obtained here should be interpreted as nominal exposure–response metrics rather than as absolute dissolved-phase toxicity thresholds.

#### 2.2.2. Toxicity Tests and Endpoint Measurements

For toxicity assays, we used 24-well microplates (30024; SPL Life Sciences, Pocheon, Gyeonggi-do, South Korea). Each well contained 0.2 mL of diatom suspension (OD_680_ = 0.5 ± 0.1) and 1.8 mL of PFAS test solution, resulting in a final volume of 2 mL. Control wells containing only DM and corresponding solvent controls were included in all experiments. Plates were incubated for 7 days under the same conditions as described for cultivation (Section 2.1).

The 7-day incubation corresponded to approximately 5–8 generations, based on the reported doubling time of *C. meneghiniana* of approximately 20–30 h [35]. This multigenerational exposure period was selected to allow detection of physiological responses that may not be apparent during conventional 72–96 h exposure periods. All assays were conducted in triplicate, and the entire experiment was independently repeated three times.

Chlorophyll fluorescence and lipid accumulation were selected as complementary endpoints to capture different but metabolically connected aspects of the physiological response of *C. meneghiniana* to chronic PFAS exposure. Chlorophyll fluorescence provides an integrated measure of chlorophyll-associated pigment status and biomass, representing the photosynthetic component of the cellular response. Lipid accumulation, in contrast, provides information on metabolic changes associated with cellular carbon and energy allocation under stress. The two endpoints were therefore combined because chronic PFAS exposure may affect photosynthetic processes and cellular metabolism differently, and a response detectable with one endpoint may not necessarily be reflected to the same extent by the other. Their simultaneous assessment within the same exposure therefore provides broader physiological coverage and improves the ability of the assay to resolve compound-specific response patterns.

##### Chlorophyll Fluorescence Measurements

After 7 days of PFAS exposure, chlorophyll a fluorescence was measured using a microplate reader (SpectraMax iD3, Molecular Devices, San Jose, CA, USA) at an excitation wavelength of 485 nm and an emission wavelength of 680 nm. This steady-state measurement reflected bulk chlorophyll fluorescence per well and was dominated by chlorophyll content across all cells in the culture volume. Accordingly, this endpoint was interpreted as an indicator of photosynthetic pigment content and overall chlorophyll-associated biomass. Background fluorescence in solvent-only controls was subtracted from the measurements of PFAS test solutions, and the resulting values were normalized to the untreated control.

##### Lipid Content Analysis by Nile Red Staining

After 7 days of PFAS exposure, the lipid content of the treated diatom cultures was quantified using Nile Red staining, adapted from Lee et al. [13]. After exposure, 297 μL of diatom suspension from each well was transferred in triplicate to a black 96-well plate. Nile Red solution (3 μL, 100 μg mL^−1^ in DMSO) was added to achieve a final concentration of 1 μg mL^−1^.

The plates were incubated in the dark at room temperature (22–25 °C) for 20 min. Fluorescence was measured using a microplate reader at an excitation wavelength of 530 nm and an emission wavelength of 592 nm. Background fluorescence in solvent-only controls was subtracted from the measurements of PFAS test solutions, and the resulting values were normalized to the untreated control.

### 2.3. Statistical Analysis

Treatment effects were analyzed separately for each PFAS and physiological endpoint using one-way analysis of variance (ANOVA) in Microsoft Excel 2024 (Microsoft Corporation, Redmond, WA, USA), with nominal PFAS concentration as the treatment factor. Each experiment was independently repeated three times, with triplicate wells included within each experimental run. To avoid treating technical replicates as independent observations, the mean of the triplicate wells within each independent experiment was used as a single independent experimental replicate (*n* = 3 per concentration).

Prior to ANOVA, the assumptions of normality and homogeneity of variance were evaluated using the Shapiro–Wilk test on model residuals and Levene’s test across concentration groups, respectively. Homogeneity of variance was satisfied for all 20 PFAS × endpoint datasets (Levene’s test, *p* > 0.05). The normality assumption was satisfied for 15 datasets but not for five datasets: PFHxS for chlorophyll fluorescence and PFBA, PFOA, PFDA, and PFUnDA for lipid accumulation. For these five datasets, the Kruskal–Wallis test was additionally performed as a non-parametric robustness check. In all cases, the Kruskal–Wallis test yielded the same conclusion regarding the significance or non-significance of the overall treatment effect as the corresponding one-way ANOVA. One-way ANOVA was therefore retained as the primary analysis for consistency across the dataset. When a significant overall treatment effect was detected (*p* < 0.05), Fisher’s least significant difference (LSD) procedure was used for pairwise comparisons among concentration groups. Half-maximal effective concentrations (EC_50_s) were estimated by linear interpolation using Toxicalc 5.0 (Tidepool Scientific, McKinleyville, CA, USA) and are reported with 95% confidence intervals. EC_50_ values represent nominal exposure concentrations and were defined as the concentrations producing a 50% change in the measured endpoint relative to the untreated control. Assay reproducibility was evaluated using the coefficient of variation (CV) of the EC_50_ estimates obtained from the three independent experiments for each PFAS and endpoint, calculated as CV (%) = SD/mean × 100.

### 2.4. Sensitivity and Reliability Analysis

Endpoint performance was evaluated using a ranking-based approach adapted from Lee et al. [13], based on EC_50_ (sensitivity) and CV (reproducibility). Smaller EC_50_ values indicate greater sensitivity, and smaller CV values indicate greater reproducibility, consistent with the use of these measures in ecotoxicological endpoint evaluation. For each PFAS and endpoint, EC_50_ values obtained independently from the three experiments for the two endpoints were ranked separately in ascending order. Thus, each criterion comprised six observations (two endpoints × three independent experiments), resulting in ranks ranging from 1 to 6, where a rank of 1 represented the lowest EC_50_ or CV and therefore the greatest sensitivity or reproducibility, respectively. When an EC_50_ could not be determined within the tested concentration range (ND), the corresponding observation was assigned rank 6 for the purpose of sensitivity ranking, representing the lowest performance within the scoring scheme. Tied values were assigned their mean rank. The sensitivity and reproducibility ranks were then summed to obtain an overall comparative performance score for each endpoint, with lower overall scores indicating better combined sensitivity and reproducibility. CVs were calculated from the three independently derived EC_50_ values for each endpoint.

### 2.5. Species Sensitivity Distribution (SSD)-Based Exploratory Comparison of Species Sensitivity

SSDs model the variability in the sensitivity of multiple species to a single toxin or other stressor. SSDs were constructed using the U.S. EPA’s SSD Toolbox (version 1.0) based on the toxicity data obtained in this study, together with published datasets for freshwater microalgae exposed to PFAS with chain lengths of C4–C11 and both functional groups. Published toxicity data were compiled from studies reporting species-specific quantitative EC_50_ values for freshwater microalgae exposed to the PFAS evaluated in the present study. Extracted information included PFAS identity, microalgal species, biological endpoint, exposure duration, EC_50_, and source reference. The literature-derived data used for SSD construction are summarized in Section 3.3.

Cumulative probability represents the proportion of species affected at or below a given concentration and corresponds to the cumulative distribution function of the fitted model applied to the log-transformed EC_50_ data.

Data were log_10_-transformed and fitted using maximum likelihood estimation. Model fit was evaluated using parametric bootstrap goodness-of-fit tests (1000 iterations) across multiple distributions (normal, logistic, triangular, Gumbel, Weibull, Burr III). Distributions with *p* > 0.05 in the parametric bootstrap goodness-of-fit test were considered to provide an adequate fit.

To ensure robustness, only datasets meeting the Canadian Environmental Protection Act minimum species criterion (*n* ≥ 7) were used for quantitative SSD derivation [36]. Model-averaged HC_5_ values were calculated by weighting candidate distributions based on their corrected Akaike Information Criterion scores.

Predicted no-effect concentrations (PNECs) were calculated as HC_5_/10, following standard assessment factors to account for uncertainty [37,38]. Quantile–quantile (Q–Q) plots were used to verify distributional fit near the HC_5_ region.

PNEC estimates are provided as exploratory values carrying substantial uncertainty. Because the SSDs combine growth-based EC_50_ values reported in the literature with the nominal, endpoint-specific values generated in this study, they are interpreted as exploratory comparisons of relative species sensitivity within broader aquatic risk assessment frameworks rather than as stand-alone regulatory thresholds.

## 3. Results

### 3.1. Structure-Dependent Toxicity of PFAS in C. meneghiniana

Chronic exposure of *C. meneghiniana* to ten structurally diverse PFAS revealed clear structure-dependent differences in physiological responses, primarily associated with carbon chain length and functional group. These responses were quantified using two endpoints: bulk chlorophyll fluorescence (reflecting chlorophyll pigment content and photosynthetic biomass per culture volume), and lipid accumulation, reflecting delayed metabolic responses.

**Figure 1:**
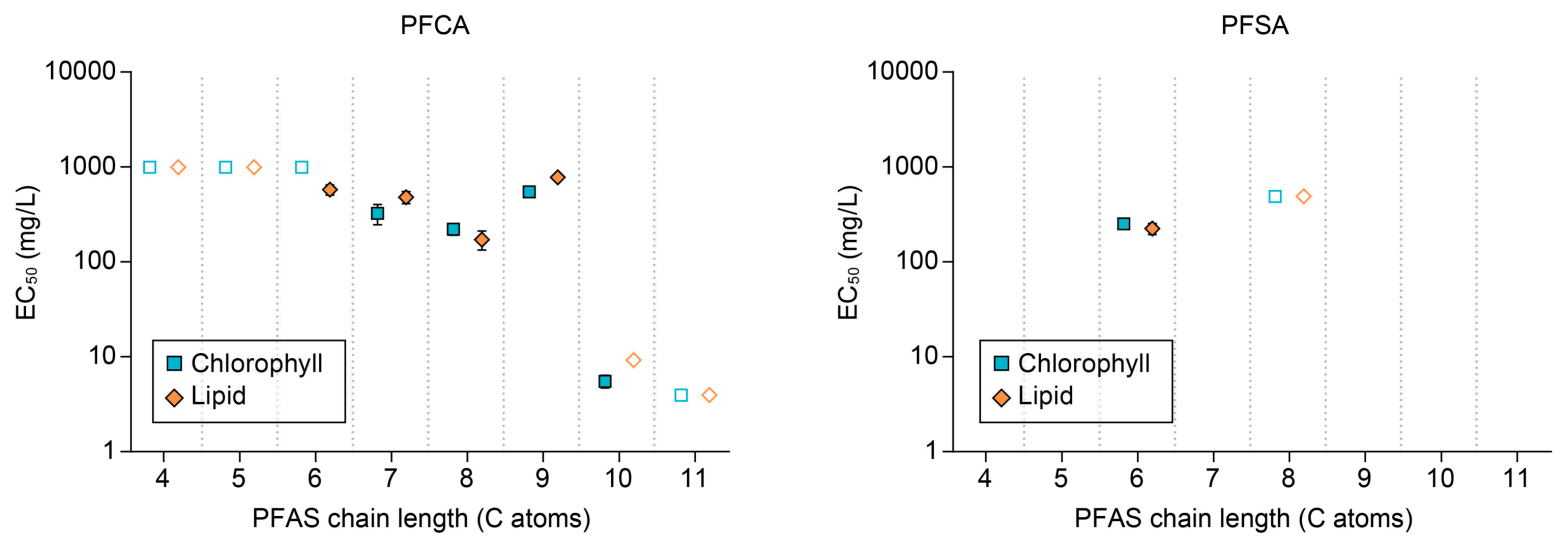
Effects of chronic PFAS exposure on photosynthesis and delayed metabolic responses in *Cyclotella meneghiniana*. EC_50_ values (mean ± standard deviation) are shown for chlorophyll fluorescence (■, blue) and lipid content (◆, orange). Error bars represent standard deviations from triplicate experiments. Open (outline-only) symbols indicate compounds for which an EC_50_ could not be determined within the tested nominal concentration range; the symbols are positioned at the highest nominal concentration tested for visualization only and do not represent quantitative EC_50_ estimates.

A pronounced chain length-dependent pattern was observed for perfluoroalkyl carboxylic acids (PFCAs). Long-chain PFCAs (C ≥ 8) significantly affected both endpoints, whereas short-chain PFCAs (C4–C5) showed no measurable effects on either endpoint up to 1000 mg L**^−^**^1^; PFHxA (C6) similarly showed no measurable fluorescence response within this range, though a lipid effect was detected (EC_50_ = 367.74 mg L^−1^) (Table 2; Figure 1). Among long-chain PFCAs, PFDA (C10) showed the strongest response among the tested PFCAs under nominal exposure conditions (fluorescence EC_50_ = 5.53 mg L^−1^) compared to PFNA (C9) (546.74 mg L^−1^), representing an approximately two orders of magnitude difference in nominal potency with a single-carbon chain extension. It should be noted, however, that the PFDA EC_50_ (5.53 mg L^−1^) falls within the dissolved concentration range (below the ~12 mg L^−1^ solubility limit), whereas the PFNA EC_50_ (546.74 mg L**^−^**^1^) was derived from test concentrations that remain well within solubility limits (~4.0 g L**^−^**^1^). The pronounced response to PFDA was one of the most striking structure-associated patterns observed in the present study. Its chlorophyll fluorescence EC_50_ (5.53 mg L^−1^) was approximately two orders of magnitude lower than that of PFNA (546.74 mg L^−1^), despite the two compounds differing by only one carbon in chain length. The PFDA EC_50_ was below its reported aqueous solubility limit (~12 mg L^−1^), suggesting that precipitation alone is unlikely to explain the pronounced PFNA–PFDA difference. Nevertheless, because dissolved exposure concentrations were not analytically verified, compound-specific differences between nominal and actual exposure cannot be excluded.

Increasing PFCA chain length is generally associated with changes in physicochemical partitioning, membrane association, and bioaccumulation potential, providing a plausible basis for stronger interactions of longer-chain compounds with cellular components. However, the magnitude of the PFNA–PFDA difference suggests that the structure–response relationship observed here cannot be explained by a simple linear increase in toxicity with chain length. Instead, the pronounced PFDA response may reflect a non-linear change in physicochemical partitioning and/or biological interaction across this region of the PFCA structural series, potentially increasing interactions with cellular membranes or photosynthetic processes and contributing to the strong chlorophyll-associated response observed for PFDA.

This interpretation remains hypothetical because dissolved exposure concentrations, intracellular PFAS accumulation, membrane partitioning, and the underlying molecular mechanisms were not directly measured. Furthermore, PFUnDA showed no measurable response within its substantially lower testable concentration range, which was constrained by its low aqueous solubility. The present data therefore do not support inference of a defined C10 threshold or a monotonic increase in toxicity beyond PFDA. Rather, the PFDA response should be regarded as a notable structure-associated discontinuity that warrants targeted investigation using analytically verified exposure concentrations and mechanistic measurements.

Differences were also observed between functional groups. For C6 compounds, PFHxS (sulfonate) showed lower EC_50_ values than PFHxA (carboxylate), particularly for fluorescence. However, this pattern was not consistent at C8: PFOS showed no measurable effect on either endpoint within the tested concentration range (up to 500 mg L**^−^**^1^), in contrast to PFOA which influenced both fluorescence and lipid accumulation. The absence of a measurable response to PFOS should not be interpreted as evidence of negligible intrinsic toxicity; rather, no quantifiable effect was detected for either endpoint within the tested nominal concentration range and experimental conditions. This distinction is particularly important given the environmental relevance and persistence of PFOS. Several factors may contribute to this observation, including compound-specific differences between sulfonates and carboxylates in physicochemical behaviour and cellular interactions, as well as endpoint-specific responsiveness. In addition, because dissolved exposure concentrations were not analytically verified, potential losses through adsorption or partitioning cannot be excluded. The PFOS result therefore highlights that PFAS toxicity in *C. meneghiniana* cannot be inferred solely from carbon-chain length and that functional group and endpoint identity may substantially influence the observed response.

These trends are illustrated in Figure 1, where EC_50_ values for PFCAs decrease with increasing chain length, while PFSA responses show greater variability. Long-chain PFCAs significantly affected both endpoints, with EC_50_ values in the range of ~5–500 mg L^−1^, whereas short-chain PFCAs showed no measurable effects within the tested concentration range.

The observed patterns were supported by generally low variability in fluorescence measurements (CV typically < 15%), indicating consistent endpoint performance across compounds.

### 3.2. Comparison of Chlorophyll Fluorescence and Lipid Accumulation Endpoints

**Figure 2:**
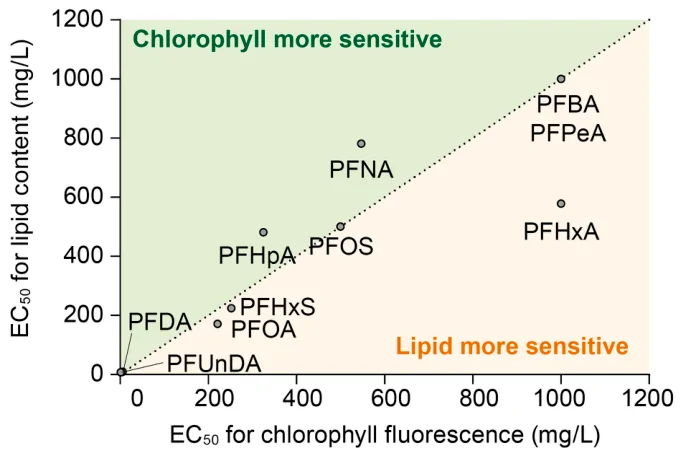
Comparison of EC_50_ values for chlorophyll fluorescence and lipid content in *Cyclotella meneghiniana* exposed to ten PFAS compounds. Each point represents a single compound, plotted according to its EC_50_ for chlorophyll fluorescence (*x*-axis) and lipid content (*y*-axis). The 1:1 reference line indicates equal sensitivity of the two endpoints. Chlorophyll fluorescence is most sensitive to compounds positioned above the line, whereas lipid content is most sensitive to those below the line.

The dual-endpoint design enabled direct comparison of sensitivity and reproducibility between chlorophyll fluorescence and lipid accumulation in response to PFAS exposure in *C. meneghiniana*. EC_50_ values for both endpoints were compared across ten PFAS compounds (Figure 2). Most compounds were distributed near the 1:1 reference line, indicating broadly comparable sensitivities between endpoints.

However, several compounds exhibited clear deviations from this pattern. For example, PFHxA showed a substantially lower EC_50_ for lipid accumulation (367.74 mg L^−1^) compared to fluorescence (>1000 mg L^−1^), indicating a stronger effect on delayed metabolic responses. In contrast, PFUnDA showed no measurable effect on either endpoint within the tested concentration range (up to 4 mg L**^−^**^1^), and EC_50_ values for both endpoints were reported as ND. The two endpoints captured distinct aspects of PFAS toxicity, and aqueous solubility limited the interpretable concentration range for certain long-chain compounds. Such deviations may arise because the two endpoints reflect different physiological processes; for example, a compound may induce metabolic carbon reallocation or lipid accumulation without producing a proportional reduction in chlorophyll fluorescence. Therefore, the marked divergence observed for PFHxA may reflect endpoint-specific physiological sensitivity rather than experimental inconsistency.

**Figure 3:**
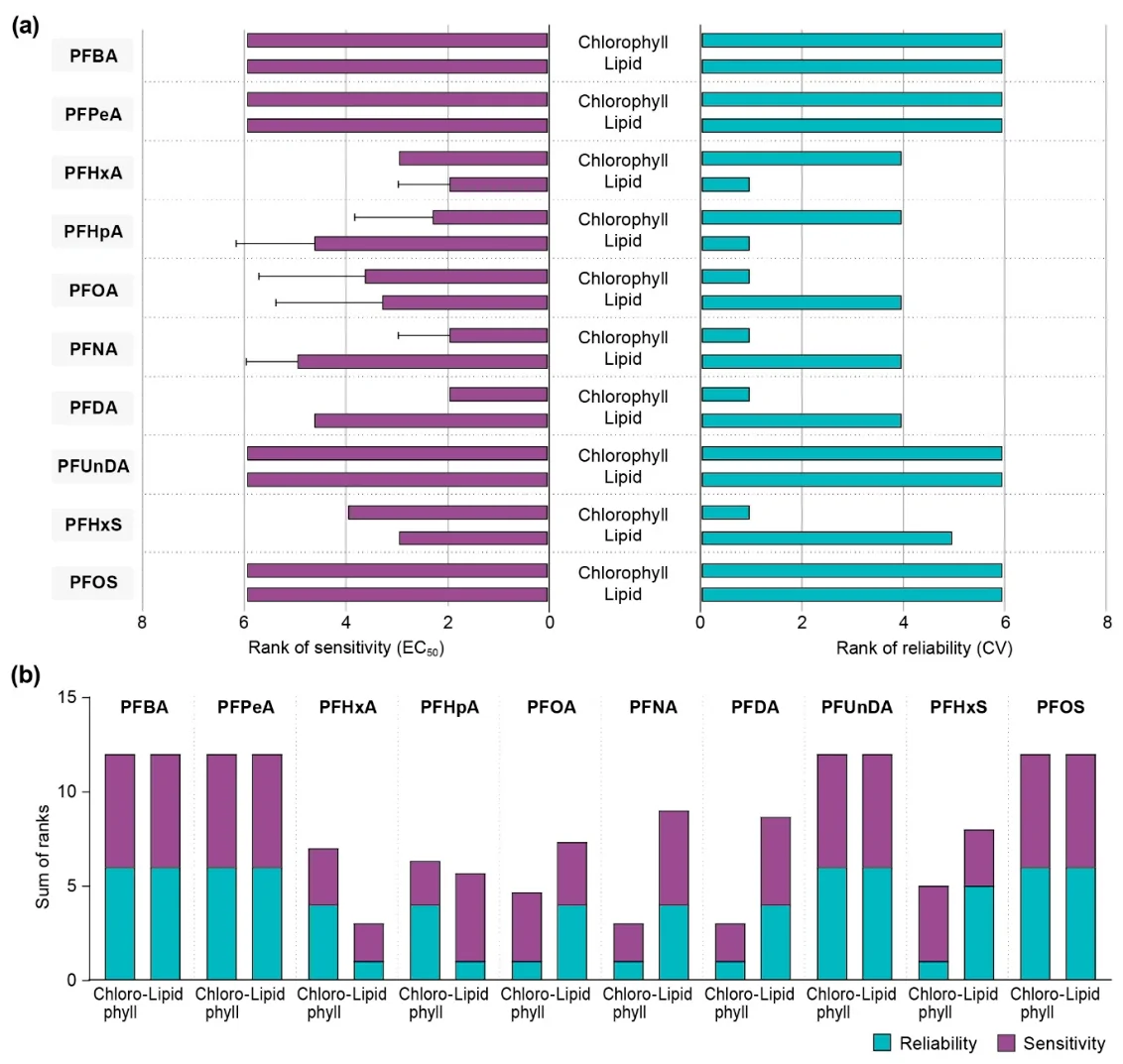
Rank-based comparison of chlorophyll fluorescence and lipid content endpoints for evaluation of PFAS toxicity. Endpoint performance was evaluated based on sensitivity (EC_50_) the effects of 10 PFAS compounds and the reproducibility (coefficient of variation, CV) of the effects; lower rank indicates better performance. (**a**) Mean (± standard deviation, *n* = 3) ranks for sensitivity and reproducibility of each endpoint for each indicated compound. (**b**) Combined performance ranking obtained as the sum of the sensitivity rank and reproducibility rank for each compound.

To quantitatively assess endpoint performance, EC_50_ values (sensitivity) and coefficients of variation (CV; reproducibility) were evaluated using a rank-based approach (Figure 3).

Chlorophyll fluorescence generally showed lower or comparable EC_50_ values relative to lipid accumulation for most compounds, and CV values were consistently low (e.g., PFHxS = 1.26%, PFNA = 2.46%), indicating high reproducibility. Lipid accumulation showed higher sensitivity for shorter-chain PFCAs (e.g., PFHxA, PFHpA), but exhibited greater variability for some compounds (e.g., PFHxS, PFOA), reflecting more heterogeneous response patterns.

When sensitivity and reproducibility were combined, chlorophyll fluorescence ranked as the more robust endpoint for four compounds (PFOA, PFNA, PFDA, and PFHxS), whereas lipid accumulation showed higher relative performance for two compounds (PFHxA and PFHpA). For PFBA, PFPeA, PFUnDA, and PFOS, no measurable effects were detected within the tested concentration ranges, and EC_50_ values could not be determined; these compounds were therefore assigned the lowest rank (6) to indicate no detectable effect and were excluded from comparative endpoint evaluation. These results indicated that neither endpoint consistently outperformed the other across all compounds, and that their combined use provided broader coverage of compound-specific PFAS responses.

### 3.3. Exploratory Species Sensitivity Distributions for Selected PFAS Compounds

To evaluate the ecological relevance of PFAS toxicity observed in this study, SSDs were constructed using EC_50_ values from *C. meneghiniana* combined with published toxicity data for freshwater microalgae (Table 3). Among the ten PFAS tested, sufficient data (*n* ≥ 7 species) for quantitative SSD analysis were available only for PFOA and PFNA. For PFOS, no measurable toxicity was detected in *C. meneghiniana* within the tested concentration range, and hence, SSD-based threshold derivation was not conducted (see Table S1). For the remaining compounds, either species data were insufficient (*n* < 7) or no measurable toxicity was detected in *C. meneghiniana* (PFBA, PFPeA, PFUnDA). Therefore, these compounds were assessed qualitatively (Table S1).

The resulting SSDs showed compound-specific differences in species sensitivity (Figure S1). For PFOA and PFNA, the derived hazardous concentrations affecting 5% of species (HC_5_) were 26,496 µg L^−1^ and 35,515 µg L^−1^, respectively, with corresponding PNECs of 2650 µg L^−1^ and 3552 µg L^−1^. These values fall within the range of previously reported algal SSD-derived thresholds. Because the underlying datasets combine growth-based EC_50_ values from the literature with the nominal, endpoint-specific values obtained here, the distributions are presented as exploratory comparisons rather than as directly comparable toxicity datasets. The full SSD outputs, comprising the model-averaged HC_5_ and PNEC estimates with their confidence intervals and the fitted distributions for all ten compounds, are provided in the Supplementary Material (Table S1 and Figure S1).

For compounds with insufficient species data (*n* < 7) or no measurable toxicity in *C. meneghiniana*, SSDs could not be reliably constructed. These results are therefore presented as qualitative indicators of relative sensitivity patterns rather than quantitative thresholds. Confidence intervals associated with HC_5_ and PNEC estimates for PFOA and PFNA were relatively wide (Table S1), reflecting both the limited number of species and variability among experimental conditions and endpoint types across studies (e.g., growth vs. photosynthesis inhibition). The SSD analysis demonstrated compound-specific variability in PFAS toxicity across microalgal species and highlighted the importance of data availability, endpoint consistency, and species coverage in deriving reliable ecotoxicological thresholds.

## 4. Discussion

In this study, we developed a chronic, multi-generational bioassay to detect sublethal effects of PFAS exposure in the freshwater diatom *C. meneghiniana*. The assay integrated bulk chlorophyll fluorescence (a steady-state measure of chlorophyll content and photosynthetic pigment status per culture volume) with lipid accumulation (an indicator of downstream metabolic adjustment associated with oxidative stress and carbon reallocation). By combining these two endpoints within a 7-day exposure framework, we resolved PFAS toxicity as a temporally structured process rather than as a single aggregated toxic outcome, thereby moving beyond conventional acute growth-based testing.

Using this framework, we identified clear structure-dependent toxicity patterns across PFAS compounds. Long-chain PFCAs induced substantially stronger effects than short-chain and sulfonated PFAS, affecting both chlorophyll fluorescence and lipid accumulation, whereas sulfonated compounds showed more selective responses. When combined with published algal datasets, SSD analysis revealed compound-specific differences in species sensitivity patterns. Hence, PFAS toxicity in microalgae was not adequately captured by single-endpoint assays, and chemical structure strongly influenced both the magnitude and the trajectory of toxicity.

Although chlorophyll fluorescence and lipid accumulation are established toxicity endpoints in microalgal studies, they have typically been applied independently or within short-term assays focused on acute responses (e.g., [28,29,54]). The structure-dependent nature of PFAS toxicity in microalgae—as a function of chain length and headgroup—is well established in the literature, as illustrated by the breadth of published data compiled in Table 3. The present study does not claim to discover this relationship. Rather, its methodological contribution lies in three respects. First, we applied both chlorophyll fluorescence and lipid accumulation within a multi-generational chronic exposure design (7 days; 5–8 generations), enabling detection of temporally separated responses that are not captured under standard short-term conditions. Second, we systematically compared the sensitivity and reproducibility of both endpoints across a structurally diverse PFAS panel. Third, we linked endpoint-specific responses to SSD-derived thresholds using a freshwater diatom, thereby connecting cellular-level dose–response data to ecologically interpretable risk metrics within a single, cohesive framework.

An important finding of the study was that long-chain PFAS, particularly long-chain PFCAs, exhibited stronger toxicity. This pattern was consistent with the greater hydrophobicity and intracellular persistence of longer-chain PFAS, which are expected to enhance their interaction with cell membranes and membrane-associated proteins [5,9]. However, hydrophobicity alone is unlikely to fully explain the observed responses. In algal systems, toxicity also depends on the extent to which the compound perturbs intracellular redox balance and triggers downstream stress responses. We found that long-chain PFCAs were not only more potent overall but were also more likely to affect both fluorescence and lipid accumulation. Hence, their effects extended beyond early reductions in photosynthetic pigment content and biomass and propagated into broader cellular metabolic disruption. This broader impact was consistent with the inference that long-chain compounds are more capable of sustaining intracellular stress and driving secondary physiological consequences. Possible contributors to the stronger responses observed for some long-chain PFCAs include stronger membrane association, greater intracellular retention, and sustained perturbation of redox and energy metabolism, which may increase the duration and magnitude of cellular stress and thereby the likelihood that photosynthetic impairment is accompanied by downstream metabolic responses. These mechanisms were not directly measured in the present study and should therefore be regarded as mechanistic interpretations supported by previous studies rather than experimentally demonstrated pathways.

PFAS toxicity in microalgae has been closely associated with oxidative stress pathways, including generation of ROS, lipid peroxidation, and disruption of photosynthetic electron transport [18,42,55]. These processes impair chloroplast function and activate compensatory responses within the cell, including antioxidant defense and metabolic reprogramming. Transcriptomic studies in diatoms support these mechanisms as they have reported up-regulation of genes involved in energy metabolism and DNA repair following PFAS exposure [18,55]. Evidence from other algal systems is consistent with this interpretation. For example, PFOA exposure increases ROS production and lipid peroxidation in *Chlorella sorokiniana* [45], while in *Nitzschia palea* it induced mitochondrial dysfunction and apoptosis-related pathways [19]. Similar responses have been reported in *Scenedesmus obliquus* and *Chlorella vulgaris*, where PFAS exposure alters antioxidant enzyme activity and increases oxidative damage [20,21,23]. Collectively, these findings support a conserved stress-response cascade in which early impairment of photosynthetic processes leads to oxidative imbalance, which in turn drives secondary metabolic adjustment.

This cascade was directly reflected in the dual-endpoint responses observed in the present study. Bulk chlorophyll fluorescence is responsive to reductions in photosynthetic biomass and pigment degradation that emerge early in the stress response [56,57]. Lipid accumulation, in contrast, reflects later-stage metabolic consequences associated with oxidative stress, membrane remodeling, and redistribution of carbon toward storage compounds such as triacylglycerols [28,29,30]. Therefore, the two endpoints in the present study did not simply provide duplicate measurements of toxicity; rather, they resolve different phases of the same stress continuum [25,56,57]. This is particularly important for PFAS, where sublethal effects may emerge progressively rather than simultaneously. The two endpoints provided complementary information on PFAS-induced physiological responses. Chlorophyll fluorescence reflected changes in chlorophyll-associated pigment status and biomass, whereas lipid accumulation reflected changes in cellular carbon and energy allocation. The greater concentration-based sensitivity of chlorophyll fluorescence for some PFAS, together with the more selective lipid responses observed across compounds, indicates that the two endpoints do not respond equivalently to PFAS exposure and therefore provide complementary physiological information [25]. These differences may be consistent with distinct stages or components of the cellular stress response, but they cannot be interpreted as evidence of a temporal sequence in the present study. Because both endpoints were measured only at the end of the 7-day exposure, the present experimental design cannot determine whether chlorophyll-associated changes precede, coincide with, or follow changes in lipid accumulation. Time-resolved measurements of both endpoints during PFAS exposure will therefore be required to establish their temporal relationships.

Differences between PFCA and PFSA responses further emphasized the importance of chemical structure. Sulfonated PFAS in this study generally exhibited weaker or more selective responses, particularly in the lipid endpoint. PFOA affected both fluorescence and lipid accumulation, whereas PFOS showed no measurable effect on either endpoint within the tested concentration range (up to 500 mg L**^−^**^1^). Consequently, SSD-based threshold derivation for PFOS could not be conducted in this study. This finding indicated that *C. meneghiniana* is relatively insensitive to PFOS compared with other microalgal species, which is consistent with the broad interspecies variability reported previously (Table 3) and highlighted the risk of underestimating PFOS hazard when algal-based benchmarks are derived from insensitive species alone. Such differences are likely to be important for interpreting PFAS toxicity at the class level because they imply that chain length and functional group chemistry jointly influence not only how strongly PFAS act but also how their effects unfold across species.

The performance characteristics of the two endpoints also have methodological significance. Chlorophyll fluorescence showed high reproducibility and consistently low coefficients of variation for most compounds, confirming its suitability as a robust quantitative endpoint for comparative toxicity testing. However, lipid accumulation showed higher relative sensitivity for shorter-chain PFCAs (PFHxA and PFHpA), indicating that neither endpoint consistently outperformed the other across all compounds. Lipid accumulation was generally less reproducible, but this lower reproducibility should not be interpreted simply as analytical weakness. Rather, it likely reflects the fact that lipid metabolism is intrinsically dynamic and integrates multiple downstream stress processes. Hence, the lipid endpoint adds interpretive value precisely because it captures a more conditional and physiologically integrative aspect of toxicity. The combined use of fluorescence and lipid accumulation strengthened the assay by providing both analytical robustness and mechanistic resolution.

Several aspects of exposure characterization warrant explicit consideration. All nominal test concentrations applied here were at or below the reported aqueous solubility limits of the corresponding compounds (Table 1), so precipitation is unlikely to have influenced the observed responses: the fluorescence EC_50_ for PFDA (5.53 mg L^−1^) lies well below its solubility limit of approximately 12 mg L^−1^, whereas for PFUnDA the highest tested concentration (4 mg L^−1^) coincided with its modelled solubility limit, which constrains the interpretable range and accounts for the absence of a measurable response. Adsorption of long-chain PFAS to polypropylene and polystyrene surfaces has also been reported in static aqueous systems and can reduce dissolved concentrations over time [58]. Although the use of identical vessels, working volumes, exposure durations, and handling procedures reduced procedural variability among treatments, it does not ensure equivalent exposure stability across PFAS. Compound-specific adsorption to test vessels, partitioning, or other losses during the 7-day exposure may have caused actual dissolved concentrations to deviate from nominal concentrations to different extents. The absence of analytical verification therefore introduces uncertainty not only into the absolute EC_50_ values but also into the quantitative magnitude of cross-compound differences and the resulting potency rankings.

Accordingly, the EC_50_ values reported here should be interpreted as nominal exposure–response metrics obtained under consistent experimental conditions rather than as analytically verified dissolved-phase toxicity thresholds. The present dataset remains useful for identifying and prioritizing structure-associated response patterns under a common screening framework, but quantitative differences among individual PFAS should be interpreted with appropriate caution. Analytical verification of exposure concentrations will be required to determine whether the magnitude of the observed cross-compound differences is retained when toxicity is expressed on the basis of measured dissolved concentrations.

The principal limitation of this study is therefore the absence of analytical verification of dissolved PFAS concentrations; PFAS uptake into diatom cells was likewise not quantified. The EC_50_ values reported here should not be interpreted as dissolved-phase toxicity thresholds, but as comparative nominal exposure–response relationships that reveal relative differences among PFAS structures under a standardized exposure design. Two further constraints apply: the mg L^−1^ concentrations required to elicit measurable responses limit direct environmental extrapolation, and the single-species design does not capture interspecific variation. Accordingly, the reported EC_50_ values should be interpreted as nominal exposure–response metrics, and broader application will require analytical confirmation of dissolved exposure concentrations, evaluation at environmentally relevant concentrations, and validation across additional microalgal species and complex environmental matrices; these steps will be necessary to determine the extent to which the proposed approach can support effect-based PFAS assessment and complement chemical monitoring.

The nominal concentrations used in the present study were substantially higher than PFAS concentrations typically reported in ambient aquatic environments, where concentrations generally occur in the ng L^−1^ range, although elevated concentrations reaching the low µg L^−1^ range have been reported at contaminated or industrially influenced sites [59,60,61]. Accordingly, the responses observed here should be interpreted primarily as comparative hazard information obtained under controlled exposure conditions rather than as evidence that equivalent effects occur under typical environmental exposure scenarios. The observed changes in chlorophyll-associated and lipid responses identify physiological processes of potential relevance to primary-producer function; however, their occurrence at environmentally relevant PFAS concentrations and their consequences at population, community, or ecosystem levels cannot be inferred from the present single-species laboratory study and remain to be established.

Reductions in chlorophyll content and photosynthetic pigment levels can constrain primary productivity at the base of aquatic food webs, while altered lipid metabolism may affect cellular energy storage and nutritional quality, with possible consequences for trophic transfer. For primary producers, these are not trivial responses: they represent changes in the way energy is captured, stored, and potentially passed on to higher trophic levels. The ecological significance of PFAS toxicity in algae lies not only in assessing whether growth is inhibited, but also in analyzing whether core physiological functions are altered in ways whose consequences under environmentally relevant PFAS exposure remain to be established.

These findings highlight important limitations in current PFAS risk assessment approaches, which still rely heavily on short-term growth inhibition endpoints. Such methods are useful for standardization, but they may fail to detect temporally structured and sublethal responses that become apparent only under longer exposure. The EC_50_ values obtained in the present study are not intended for direct comparison with environmental concentrations; instead, they provide dose–response information that can be integrated into SSD analysis and can help derive PNECs. Measured PFAS concentrations in surface waters typically range from the ng L**^−^**^1^ to low μg L**^−^**^1^ level, far below the mg L**^−^**^1^ test concentrations used here. Current regulatory benchmarks—such as the EU Environmental Quality Standard of 0.65 ng L**^−^**^1^ for PFOS [6] and the U.S. EPA maximum contaminant level of 4 ng L**^−^**^1^ for PFOA/PFOS [4]—are six to nine orders of magnitude below the EC_50_ values reported in this study. The EC_50_ values derived here are not measures of environmental risk at prevailing contamination levels but they characterize the intrinsic hazard potential and relative potency of each compound and serve as inputs to SSD modelling for derivation of algal-based thresholds. Hence, the value of chronic bioassays lies in generating biologically resolved response data that can be scaled into ecological risk metrics.

From a regulatory perspective, these results may, following further validation, help inform the integration of chronic, multi-endpoint bioassays into existing assessment frameworks. Chlorophyll fluorescence is readily compatible with standardized protocols such as OECD 201 and ISO 8692, while lipid-based endpoints add mechanistic resolution that improves interpretation of chronic toxicity. Incorporating both endpoints within extended exposure designs would allow regulatory testing to capture both early and delayed responses to PFAS exposure. This is especially relevant for contaminants such as PFAS, whose persistence and complex structure–activity relationships make acute single-endpoint assays insufficient as stand-alone tools. From a water-quality assessment perspective, the present assay should be regarded as a proof-of-concept effect-based screening approach rather than a method ready for regulatory implementation. Its potential value lies in providing biological-response information that may, following further validation, complement concentration-based chemical monitoring. The dual-endpoint design may be useful for identifying differences in biological activity among PFAS or PFAS-containing samples that are not apparent from chemical concentration data alone. However, the present study was conducted with individual PFAS under controlled laboratory conditions and does not establish performance for environmental mixtures, treated waters, or field samples. Application to water-quality assessment will therefore require validation across additional primary-producer species, environmentally relevant exposure concentrations, analytically verified exposures, PFAS mixtures, and complex environmental matrices.

The SSD results reinforced this point by showing that algal-based thresholds provide compound-dependent insights into PFAS toxicity. For PFCAs, algal SSD-derived thresholds aligned reasonably well with broader sensitivity patterns, whereas for PFSAs, they may not fully capture the sensitivity of more responsive taxa. Hence, while Algal SSDs are valuable, but they should be interpreted as complementary rather than exhaustive. Their strength lies in resolving primary producer responses and in revealing structure-dependent patterns that might otherwise be masked in broader datasets. Their limitation is that they cannot, by themselves, represent the full diversity of sensitivity across aquatic communities. Additionally, SSD-derived values are uncertain, particularly when species coverage is limited and studies differ in endpoint type or experimental design. Hence, the thresholds derived in this study should be treated as indicative metrics for comparative interpretation rather than definitive stand-alone regulatory values.

The structure-dependent patterns observed in the study suggest that PFAS risk assessment should not rely solely on single-compound thresholds. Class-based approaches that consider chain length and functional group effects may provide a more realistic basis for hazard evaluation. In particular, the low apparent toxicity of short-chain PFAS in acute assays should not be interpreted as low environmental risk because their persistence supports prolonged environmental exposure and their mobility facilitates widespread dispersion across aquatic systems, increasing the likelihood of chronic and spatially extensive impacts. Thus, low acute potency may not necessarily translate into low ecological concern.

Taken together, this study provides a proof-of-concept screening platform for rapidly identifying structure-dependent PFAS toxicity using a dual-endpoint diatom assay. Its value lies less in the absolute toxicity values generated than in its capacity to resolve, within a single standardized 7-day test, how chain length and headgroup chemistry jointly determine which physiological process is affected, at what relative potency, and in what order. This positions the assay as a first-tier screening tool for the large number of structurally diverse PFAS that remain toxicologically uncharacterized, with analytically verified confirmatory testing reserved for the compounds that the screen identifies as most potent.

## 5. Conclusions

Chronic PFAS exposure in *C. meneghiniana* produced complementary chlorophyll-associated and lipid responses that were not fully represented by either endpoint alone. By integrating chlorophyll fluorescence and lipid accumulation within a 7-day multigenerational framework, the proposed assay provided complementary information on photophysiological and metabolic responses to PFAS exposure. Because both endpoints were measured only at the end of the exposure period, however, their temporal ordering cannot be determined from the present study.

The nominal test concentrations used here were substantially higher than PFAS concentrations typically reported in ambient aquatic environments, and dissolved exposure concentrations were not analytically verified. Accordingly, the EC_50_ values should be interpreted as nominal exposure–response metrics for comparative screening under controlled experimental conditions rather than as dissolved-phase toxicity thresholds or measures of direct environmental risk. Within these constraints, the PFAS panel revealed distinct structure-associated response patterns. Among the tested PFCAs, nominal potency generally increased with chain length, with a particularly pronounced difference between PFNA (C9) and PFDA (C10), although the limited testable concentration range for PFUnDA precludes inference of a monotonic chain-length relationship or a defined C10 threshold. PFCAs and PFSAs also differed in their response patterns, and chlorophyll fluorescence and lipid accumulation showed compound-dependent differences in sensitivity, supporting the value of their combined use for resolving complementary physiological responses.

Overall, the dual-endpoint *C. meneghiniana* assay provides a proof-of-concept framework for comparative PFAS screening under controlled laboratory conditions. Its present value lies in identifying and prioritizing structure-associated biological response patterns rather than providing regulatory toxicity thresholds or predicting ecosystem-level effects. Further development will require analytical verification of exposure concentrations, preferably at multiple points during the chronic exposure period, together with testing at environmentally relevant concentrations, validation across additional microalgal species, and evaluation using PFAS mixtures and complex environmental matrices. Where feasible, cellular uptake measurements would further strengthen interpretation of the relationship between external exposure and biological response. Such validation will be necessary before the assay can be considered for broader effect-based environmental assessment.

## Figures and Tables

**Table 1:** Structural and physicochemical characteristics of the ten PFAS used to evaluate structure-dependent toxicity in *Cyclotella meneghiniana*.

Compound	Abbreviation	Carbon Chain Length	Functional Group	Category	CAS No.	Molecular Formula
Perfluorobutanoic acid	PFBA	C4	Carboxylate (PFCA)	Short-chain	375-22-4	C_4_HF_7_O_2_
Perfluoropentanoic acid	PFPeA	C5	Carboxylate (PFCA)	Short-chain	2706-90-3	C_5_HF_9_O_2_
Perfluorohexanoic acid	PFHxA	C6	Carboxylate (PFCA)	Short-chain	307-24-4	C_6_HF_11_O_2_
Perfluoroheptanoic acid	PFHpA	C7	Carboxylate (PFCA)	Mid-chain	375-85-9	C_7_HF_13_O_2_
Perfluorooctanoic acid	PFOA	C8	Carboxylate (PFCA)	Long-chain	335-67-1	C_8_HF_15_O_2_
Perfluorononanoic acid	PFNA	C9	Carboxylate (PFCA)	Long-chain	375-95-1	C_9_HF_17_O_2_
Perfluorodecanoic acid	PFDA	C10	Carboxylate (PFCA)	Long-chain	335-76-2	C_10_HF_19_O_2_
Perfluoroundecanoic acid	PFUnDA	C11	Carboxylate (PFCA)	Long-chain	2058-94-8	C_11_HF_21_O_2_
Perfluorohexane sulfonate	PFHxS	C6	Sulfonate (PFSA)	Short-chain	355-46-4	C_6_HF_13_O_3_S
Perfluorooctane sulfonate	PFOS	C8	Sulfonate (PFSA)	Long-chain	1763-23-1	C_8_HF_17_O_3_S

Note on aqueous solubility: published water solubility values (experimental and modelled; sources: PubChem) for the ten PFAS tested are as follows (approximate values at ambient temperature): PFBA ~1000 mg L^−1^; PFPeA ~1000 mg L^−1^; PFHxA ~1000 mg L^−1^; PFHpA ~500 mg L^−1^; PFOA ~3.4 g L^−1^; PFNA ~4.0 g L^−1^; PFDA ~12 mg L^−1^; PFUnDA ~4 mg L^−1^ (modelled, PubChem); PFHxS ~519 mg L^−1^; PFOS ~519 mg L^−1^ (OECD, 2002). For PFOS and PFUnDA, no measurable effects were detected within the tested concentration ranges and EC_50_ values are reported as ND. For PFDA, the derived EC_50_ (5.53 mg L^−1^) falls below its solubility limit (~12 mg L^−1^) and falls below the reported aqueous solubility limit (~12 mg L^−1^), reducing concern regarding precipitation; however, the value remains a nominal exposure estimate because dissolved concentrations were not analytically verified. All nominal test concentrations applied in this study were at or below the reported aqueous solubility limit of the corresponding compound, so loss of test substance through precipitation is unlikely. Exposure concentrations are reported as nominal values; because all compounds were tested under identical conditions, the resulting dataset supports comparative (relative) rather than absolute toxicity interpretation (Section 2.2.1 and Section 4).

**Table 2:** Effects of chronic PFAS exposure on photosynthetic and metabolic endpoints in *Cyclotella meneghiniana*. EC_50_ values are based on nominal exposure concentrations. Estimated log K_ow_ values are approximate literature-derived values. Open-ended values indicate that the EC_50_ exceeded the highest nominal concentration tested. ND, not determined; CV, coefficient of variation; PFCA, perfluoroalkyl carboxylic acid; PFSA, perfluoroalkyl sulfonic acid.

Compound	Chain Length	Category	Functional Group	Estimated Log K_ow_	Nominal Fluorescence EC_50_ (mg L^−1^)	CV (%)	Nominal Lipid EC_50_ (mg L^−1^)	CV (%)
PFBA	C4	Short	PFCA	0.5–1.0	>1000	ND	>1000	ND
PFPeA	C5	Short	PFCA	1.0–1.5	>1000	ND	>1000	ND
PFHxA	C6	Short	PFCA	1.5–2.0	>1000	ND	367.74	19.35
PFHpA	C7	Mid	PFCA	2.0–2.5	324.97	24.02	481.23	14.39
PFOA	C8	Long	PFCA	2.5–3.0	220.79	13.61	171.89	22.72
PFNA	C9	Long	PFCA	3.0–3.5	546.74	2.46	671.76	4.91
PFDA	C10	Long	PFCA	3.5–4.0	5.53	15.29	>10	ND
PFUnDA	C11	Long	PFCA	4.0–4.5	>4	ND	>4	ND
PFHxS	C6	Short	PFSA	2.0–2.5	252.28	1.26	224.46	13.21
PFOS	C8	Long	PFSA	3.0–4.0	>500	ND	>500	ND

**Table 3:** Comparison of PFAS toxicity in *Cyclotella meneghiniana* with published toxicity data for freshwater microalgae. EC_50_ values from the present study are based on nominal exposure concentrations, and literature ranges represent values reported across different species and endpoints. FL, fluorescence; LP, lipid; GR, growth; PS, photosynthesis; ND, not determined. *T. pseudonana*, *Thalassiosira pseudonana*; *R. subcapitata*, *Raphidocelis subcapitata*; *S. obliquus*, *Scenedesmus obliquus*; *C. vulgaris*, *Chlorella vulgaris*.

Compound	This Study (*C. meneghiniana*; mg L^−1^)		Reported Range (Other Microalgae) (mg L^−1^)	Most Sensitive Species (Literature)	Endpoint Type	References
	FL	LP				
PFBA	>1000	>1000	15–290	*T. pseudonana*	GR, PS	[18,26,39,40]
PFPeA	>1000	>1000	18–341	*T. pseudonana*	GR	[18,26,40]
PFHxA	>1000	367.74	18–1484	*T. pseudonana*	GR	[18,40,41,42]
PFHpA	324.97	481.23	440–1900	*R. subcapitata*	GR	[18,41]
PFOA	220.79	171.89	13–978	*T. pseudonana*	GR	[18,19,22,23,24,39,41,43,44,45,46,47,48,49]
PFNA	546.74	671.76	44–497	*S. obliquus*	GR	[22,24,39,40,41]
PFDA	5.53	>10	26–386	*R. subcapitata*	GR	[39,40]
PFUnDA	>4	>4	ND	ND	–	
PFHxS	252.28	224.46	ND	ND	–	
PFOS	>500	>500	8–320	*C. vulgaris*	GR	[24,40,41,42,43,46,47,50,51,52,53]

## Data Availability

The original contributions presented in this study are included in the article/Supplementary Material. Further inquiries can be directed to the corresponding author.

## Supplementary Materials

The following supporting information can be downloaded at https://www.mdpi.com/article/10.3390/jox16040155/s1, Figure S1: Species sensitivity distributions (SSDs) for selected PFAS compounds.; Table S1: SSD-derived hazardous concentrations affecting 5% of species (HC_5_) and predicted no-effect concentrations (PNECs) for PFAS effects on freshwater microalgae.

## References

[1] Bartley M.C., Tremblay T., De Silva A.O., Kamula C.M., Ciastek S., Kuzyk Z.Z.A. (2024). Sedimentary records of contaminant inputs in Frobisher Bay, Nunavut. Environ. Sci. Ecotechnol..

[2] Hussain H.N., Jilani M.I., Imtiaz F., Ahmed T., Arshad M.B., Mudassar M., Sharif M.N. (2025). Advances in the removal of polyfluoroalkyl substances (PFAS) from water using destructive and non-destructive methods. Green Anal. Chem..

[3] Banyoi S.-M., Porseryd T., Larsson J., Grahn M., Dinnétz P. (2022). The effects of exposure to environmentally relevant PFAS concentrations for aquatic organisms at different consumer trophic levels: Systematic review and meta-analyses. Environ. Pollut..

[4] U.S. Environmental Protection Agency (USEPA) (2023). Final PFAS National Primary Drinking Water Regulation.

[5] Stockholm Convention Secretariat (2022). Listing of Perfluorohexane Sulfonic Acid (PFHxS), Its Salts and PFHxS-Related Compounds in Annex A of the Stockholm Convention (Decision SC-10/13, Adopted at COP-10, 2022).

[6] European Union (2013). Directive 2013/39/EU of the European Parliament and of the Council of 12 August 2013 amending Directives 2000/60/EC and 2008/105/EC as regards priority substances in the field of water policy. Off. J. Eur. Union.

[7] Glüge J., Scheringer M., Cousins I.T., DeWitt J.C., Goldenman G., Herzke D., Lohmann R., Ng C.A., Trier X., Wang Z. (2020). An overview of the uses of per- and polyfluoroalkyl substances (PFAS). Environ. Sci. Process. Impacts.

[8] Hong M.-S., Lee J.-S., Lee M.-C., Lee J.-S. (2025). Ecotoxicological effects of per- and polyfluoroalkyl substances in aquatic organisms: A review. Mar. Pollut. Bull..

[9] Ahrens L., Bundschuh M. (2014). Fate and effects of poly- and perfluoroalkyl substances in the aquatic environment: A review. Environ. Toxicol. Chem..

[10] Ankley G.T., Cureton P., Hoke R.A., Houde M., Kumar A., Kurias J., Lanno R., McCarthy C., Newsted J., Salice C.J. (2021). Assessing the ecological risks of per- and polyfluoroalkyl substances: Current state of the science and a proposed path forward. Environ. Toxicol. Chem..

[11] Brunn H., Arnold G., Körner W., Rippen G., Steinhäuser K.G., Valentin I. (2023). PFAS: Forever chemicals—Persistent, bioaccumulative and mobile. Reviewing the status and the need for their phase out and remediation of contaminated sites. Environ. Sci. Eur..

[12] Kwiatkowski C.F., Andrews D.Q., Birnbaum L.S., Bruton T.A., DeWitt J.C., Knappe D.R., Maffini M.V., Miller M.F., Pelch K.E., Reade A. (2020). Scientific basis for managing PFAS as a chemical class. Environ. Sci. Technol. Lett..

[13] Lee H., Nguyen D.-V., Wu D., De Saeger J., Park M., Lee S.D., Yu Y., Lee J., Lee C., Han T. (2024). A rapid and multi-endpoint ecotoxicological test using *Mychonastes afer* for efficient screening of metals and herbicides. Ecotoxicol. Environ. Saf..

[14] (2012). Water Quality—Fresh Water Algal Growth Inhibition Test with Unicellular Green Algae.

[15] Organisation for Economic Co-operation and Development (OECD) (2011). Test No. 201: Freshwater Alga and Cyanobacteria, Growth Inhibition Test.

[16] Park J., Lee H., Asselman J., Janssen C., Depuydt S., De Saeger J., Friedl T., Sabbe K., Vyverman W., Philippart C.J.M. (2024). Harnessing the power of tidal flat diatoms to combat climate change. Crit. Rev. Environ. Sci. Technol..

[17] Pandey L.K., Bergey E.A., Lyu J., Park J., Choi S., Lee H., Depuydt S., Oh Y.-T., Lee S.-M., Han T. (2017). The use of diatoms in ecotoxicology and bioassessment: Insights, advances and challenges. Water Res..

[18] Shi J., Hu M., Xia Z., Zhang J., Wang Z., Li L., Zhao Y. (2024). Influence of perfluoroalkyl substances, with focus on perfluorobutanoic acid, on the responding characteristics and molecular mechanisms of *Thalassiosira pseudonana*. Ecotoxicol. Environ. Saf..

[19] Zhu Q., Asimtul P., Bai J., Sui F., Lu X., Mei X., Liu Y., Jiang C., Wang X., Song C. (2025). Toxic effects of PFOA on *Nitzschia palea* and its apoptosis mechanism. Algal Res..

[20] Xu D., Chen X., Shao B. (2017). Oxidative damage and cytotoxicity of perfluorooctane sulfonate on *Chlorella vulgaris*. Bull. Environ. Contam. Toxicol..

[21] Xue X., Gao N., Xu F. (2022). Toxicity of perfluorooctane sulfonate (PFOS) and perfluorobutane sulfonate (PFBS) to *Scenedesmus obliquus*: Photosynthetic characteristics, oxidative damage and transcriptome analysis. Environ. Pollut..

[22] Hu C., Luo Q., Huang Q. (2014). Ecotoxicological effects of perfluorooctanoic acid on freshwater microalgae *Chlamydomonas reinhardtii* and *Scenedesmus obliquus*. Environ. Toxicol. Chem..

[23] Xu D., Li C., Chen H., Shao B. (2013). Cellular response of freshwater green algae to perfluorooctanoic acid toxicity. Ecotoxicol. Environ. Saf..

[24] Mao W., Li M., Xue X., Cao W., Wang X., Xu F., Jiang W. (2023). Bioaccumulation and toxicity of perfluorooctanoic acid and perfluorooctane sulfonate in marine algae *Chlorella* sp. Sci. Total Environ..

[25] Shi T.-Q., Wang L.-R., Zhang Z.-X., Sun X.-M., Huang H. (2020). Stresses as first-line tools for enhancing lipid and carotenoid production in microalgae. Front. Bioeng. Biotechnol..

[26] Flynn K.M., Bush K., Cavallin J., Hazemi M., Kasparek A., Schumann P., Villeneuve D.L. (2025). Transcriptomic response of an algal species (*Raphidocelis subcapitata*) exposed to 22 per- and polyfluoroalkyl substances. Environ. Toxicol. Chem..

[27] Soares L.O.S., de Araujo G.F., Gomes T.B., Júnior S.F.S., Cuprys A.K., Soares R.M., Saggioro E.M. (2025). Antioxidant system alterations and oxidative stress caused by polyfluoroalkyl substances (PFAS) in exposed biota: A review. Sci. Total Environ..

[28] Du Z.-Y., Benning C., Nakamura Y., Li-Beisson Y. (2016). Triacylglycerol accumulation in photosynthetic cells in plants and algae. Lipids in Plant and Algae Development.

[29] Juergens M.T., Disbrow B., Shachar-Hill Y. (2016). The relationship of triacylglycerol and starch accumulation to carbon and energy flows during nutrient deprivation in *Chlamydomonas reinhardtii*. Plant Physiol..

[30] Li-Beisson Y., Roston R.L. (2024). Plant and Algal Lipids: In All Their States and on All Scales. Plant Cell Physiol..

[31] de Haan L.R., Reiniers M.J., Reeskamp L.F., Belkouz A., Ao L., Cheng S., Ding B., van Golen R.F., Heger M. (2022). Experimental conditions that influence the utility of 2′,7′-dichlorodihydrofluorescein diacetate (DCFH_2_-DA) as a fluorogenic biosensor for mitochondrial redox status. Antioxidants.

[32] Gao Q.T., Wong Y.S., Tam N.F.Y. (2017). Antioxidant responses of different microalgal species to nonylphenol-induced oxidative stress. J. Appl. Phycol..

[33] Beakes G.W., Canter H.M., Jaworski G.H. (1988). Zoospore ultrastructure of *Zygorhizidium affluens* and *Z. planktonicum*, two chytrids parasitizing the diatom *Asterionella formosa*. Can. J. Bot..

[34] National Institute of Environmental Research (NIER) (2024). Unregulated Water Pollutants Screening Program (2024).

[35] Graham J.M., Graham L.E., Zulkifly S.B., Pfleger B.F., Hoover S.W., Yoshitani J. (2012). Freshwater diatoms as a source of lipids for biofuels. J. Ind. Microbiol. Biotechnol..

[36] Government of Canada (2023). Species Sensitivity Distributions for Water Quality Guidelines and Ecological Risk Assessment.

[37] de Groot-Heijtel C., van Vlaardingen P., Aldenberg T., Smit C., Verbruggen E., Kraak M. (2024). Derivation of environmental quality standards for free cyanide incorporating censored data into species sensitivity distributions. Sci. Total Environ..

[38] Lapczynski A., Belanger S.E., Connors K., Bozich J. (2024). A chronic aquatic hazard assessment for the perfume raw material octahydro-tetramethyl-naphthalenyl-ethanone. Environ. Toxicol. Chem..

[39] Li X., Zhang P., Jin L., Shao T., Li Z., Cao J. (2012). Efficient photocatalytic decomposition of perfluorooctanoic acid by indium oxide and its mechanism. Environ. Sci. Technol..

[40] Pietropoli E., Bardhi A., Simonato V., Zanella M., Iori S., Barbarossa A., Giantin M., Dacasto M., De Liguoro M., Pauletto M. (2024). Comparative toxicity assessment of alternative versus legacy PFAS: Implications for two primary trophic levels in freshwater ecosystems. J. Hazard. Mater..

[41] Latała A., Nędzi M., Stepnowski P. (2009). Acute toxicity assessment of perfluorinated carboxylic acids towards the Baltic microalgae. Environ. Toxicol. Pharmacol..

[42] Ma T., Ye C., Wang T., Li X., Luo Y. (2022). Toxicity of per- and polyfluoroalkyl substances to aquatic invertebrates, plankton, and microorganisms. Int. J. Environ. Res. Public Health.

[43] Rodea-Palomares I., Gonzalo S., Santiago-Morales J., Leganés F., García-Calvo E., Rosal R., Fernández-Piñas F. (2012). An insight into the mechanisms of nanoceria toxicity in aquatic photosynthetic organisms. Aquat. Toxicol..

[44] Rosal R., Rodea-Palomares I., Boltes K., Fernández-Piñas F., Leganés F., Petre A. (2010). Ecotoxicological assessment of surfactants in the aquatic environment: Combined toxicity of docusate sodium with chlorinated pollutants. Chemosphere.

[45] Zhao Z., Zheng X., Han Z., Yang S., Zhang H., Lin T., Zhou C. (2023). Response mechanisms of *Chlorella sorokiniana* to microplastics and PFOA stress: Photosynthesis, oxidative stress, extracellular polymeric substances and antioxidant system. Chemosphere.

[46] Ma W., Zhao M., Zhao J., Guo Z., Cui Y., Li H. (2022). Preparation of superamphiphobic stainless steel surface by laser and perfluorooctanoic acid treatment. Opt. Laser Technol..

[47] Mhadhbi L., Rial D., Pérez S., Beiras R. (2012). Ecological risk assessment of perfluorooctanoic acid (PFOA) and perfluorooctanesulfonic acid (PFOS) in the marine environment using *Isochrysis galbana*, *Paracentrotus lividus*, *Siriella armata* and *Psetta maxima*. J. Environ. Monit..

[48] González-Naranjo V., Boltes K. (2014). Toxicity of ibuprofen and perfluorooctanoic acid for risk assessment of mixtures in aquatic and terrestrial environments. Int. J. Environ. Sci. Technol..

[49] Mojiri A., Nazari Vishkaei M., Ansari H.K., Vakili M., Farraji H., Kasmuri N. (2023). Toxicity effects of perfluorooctanoic acid (PFOA) and perfluorooctane sulfonate (PFOS) on two green microalgae species. Int. J. Mol. Sci..

[50] Zhang D.-Y., Xu X.-L., Shen X.-Y. (2012). Effects of perfluorooctane sulfonate (PFOS) on physiological status and proliferation capacity of *Chlorella pyrenoidosa*. Proceedings of the 2012 International Conference on Biomedical Engineering and Biotechnology (ICBEB), Macau, China, 28–30 May 2012.

[51] Boudreau T., Sibley P., Mabury S., Muir D., Solomon K. (2003). Laboratory evaluation of the toxicity of perfluorooctane sulfonate (PFOS) on *Selenastrum capricornutum*, *Chlorella vulgaris*, *Lemna gibba*, *Daphnia magna* and *Daphnia pulicaria*. Arch. Environ. Contam. Toxicol..

[52] Mitchell R.J., Myers A.L., Mabury S.A., Solomon K.R., Sibley P.K. (2011). Toxicity of fluorotelomer carboxylic acids to the algae *Pseudokirchneriella subcapitata* and *Chlorella vulgaris*, and the amphipod *Hyalella azteca*. Ecotoxicol. Environ. Saf..

[53] Phillips M.M., Dinglasan-Panlilio M.J.A., Mabury S.A., Solomon K.R., Sibley P.K. (2007). Fluorotelomer acids are more toxic than perfluorinated acids. Environ. Sci. Technol..

[54] Sjollema S.B., van Beusekom S.A.M., van der Geest H.G., Booij P., de Zwart D., Vethaak A.D., Admiraal W. (2014). Laboratory algal bioassays using PAM fluorometry: Effects of test conditions on the determination of herbicide and field sample toxicity. Environ. Toxicol. Chem..

[55] Liu Z., Cao X., Wu M., Huang W., Dong X., Chen X., Zhang C. (2025). Mechanisms of PFBA toxicity in *Chlorella vulgaris*: Photosynthesis, oxidative stress and antioxidant impairment. Environ. Res..

[56] Chen C.-I., Lin K.-H., Lin T.-C., Huang M.-Y., Chen Y.-C., Huang C.-C., Wang C.-W. (2023). Responses of photosynthesis and chlorophyll fluorescence during light induction in different seedling ages of *Mahonia oiwakensis*. Bot. Stud..

[57] Fitzgerald N.J.M., Wargenau A., Sorenson C., Pedersen J., Tufenkji N., Novak P.J., Simcik M.F. (2018). Partitioning and accumulation of perfluoroalkyl substances in model lipid bilayers and bacteria. Environ. Sci. Technol..

[58] Zenobio J.E., Salawu O.A., Han Z., Adeleye A.S. (2022). Adsorption of per- and polyfluoroalkyl substances (PFAS) to containers. J. Hazard. Mater. Adv..

[59] Gleason J.A., Cooper K.R., Klotz J.B., Post G.B., Van Orden G. (2015). Health-Based Maximum Contaminant Level Support Document: Perfluorononanoic Acid (PFNA).

[60] Wee S.Y., Aris A.Z. (2023). Revisiting the “forever chemicals”, PFOA and PFOS exposure in drinking water. npj Clean Water.

[61] Post G.B., Louis J.B., Lippincott R.L., Procopio N.A. (2013). Occurrence of perfluorinated compounds in raw water from New Jersey public drinking water systems. Environ. Sci. Technol..

