# Supplementary material for: Nominal Concentration-Based Assessment of Structure-Dependent PFAS Responses in the Freshwater Diatom Cyclotella meneghiniana Using Dual Physiological Endpoints
Source: J Xenobiot. 2026 Aug 21;16(4):155. doi: 10.3390/jox16040155 (PMC13514860; doi:10.3390/jox16040155)

Supplementary Materials: Nominal Concentration-Based Assessment of Structure-Dependent PFAS Responses in the Freshwater Diatom *Cyclotella meneghiniana* Using Dual Physiological Endpoints

Hojun Lee, Taejun Han and Jihae Park

Table S1. SSD-derived hazardous concentrations affecting 5% of species (HC$_5$) and predicted no-effect concentrations (PNECs) for PFAS effects on freshwater microalgae. Only datasets meeting the minimum species requirement (n ≥ 7) were used for quantitative SSD analysis. Confidence intervals (CI) reflect model uncertainty due to limited data availability and inter-study variability. These values are intended for comparison only; they should not be used as stand-alone regulatory thresholds. †PFOS: no measurable toxicity was detected in *Cyclotella meneghiniana* within the tested concentration range; SSD-based threshold derivation was therefore not conducted in this study.

Compound	No. of species	HC$_5$ (μg L^{-1})	95% CI	PNEC (μg L^{-1})	95% CI
PFOA	17	26,496	14,355–53,037	2,650	1,436–5,304
PFNA	10	35,515	16,646–94,160	3,552	1,665–9,416
PFBA	5	ND	–	ND	–
PFPeA	3	ND	–	ND	–
PFHxA	6	ND	–	ND	–
PFHpA	5	ND	–	ND	–
PFDA	3	ND	–	ND	–
PFUnDA	1	ND	–	ND	–
PFHxS	1	ND	–	ND	–
PFOS†	–	ND	–	ND	–

Figure S1. Species sensitivity distributions (SSDs) for selected PFAS compounds. EC$_{50}$ toxicity values from this study on *Cyclotella meneghiniana* and published toxicity data on freshwater microalgae were used for the analysis. The curves show model-averaged fits of log-transformed toxicity data. Cumulative probability represents the proportion of species affected at or below a given concentration. Shaded areas indicate 95% confidence intervals. The red lines indicate hazardous concentrations affecting 5% of species (HC$_5$). The species for which data was analysed are indicated. A minimum of seven species is requirement for SSD analysis; the plots with fewer than seven species indicated are presented as complementary evidence to support interpretation of structure-dependent toxicity, rather than as stand-alone regulatory thresholds.

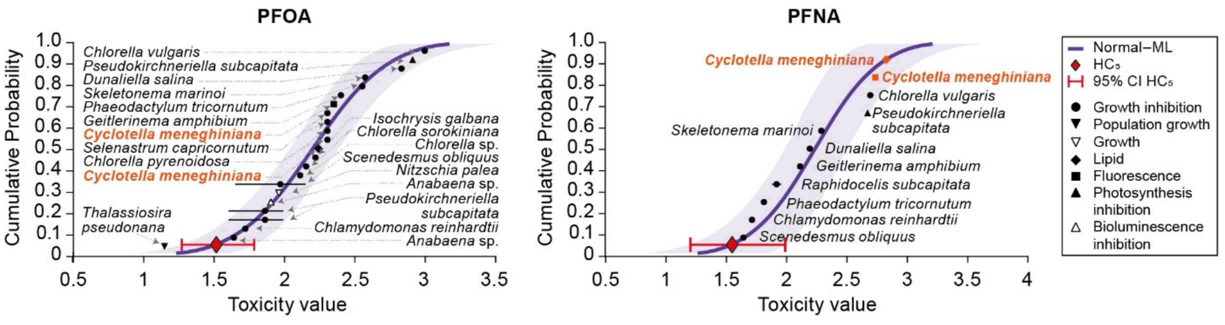

Supplement: Supplementary file 1 [file jox-16-00155-s001.zip › jox-4508487-supplementary.pdf]
